# Fathers’ involvement in complementary feeding of children in Damot Woyde District, South Ethiopia: a community-based cross-sectional study

**DOI:** 10.1186/s40795-023-00670-8

**Published:** 2023-01-10

**Authors:** Amare Abebe Wolkanto, Tsegaye Demissie Gemebo, Samson Kastro Dake, Tewodros Getachew Hailemariam

**Affiliations:** 1 Department of Reproductive Health and Nutrition, School of Public Health, College of Health Sciences and Medicine, Wolaita Sodo University, Wolaita Sodo, Ethiopia; 2 New South Wales Cancer Institute, Sydney, Australia

**Keywords:** Children, Complementary feeding, Fathers’ involvement, 6–23 months

## Abstract

**Background:**

In infant and young child feeding practice parents are the primary agents for childcare activities, such as feeding. Mothers’ role in infant and young child feeding practice has been the focus of previous research. The involvement of fathers in child-feeding practice has rarely been studied. Thus, this study aimed to assess fathers’ involvement in the complementary feeding of children and identify factors associated with it in Southern Ethiopia.

**Methods:**

A community-based cross-sectional study was conducted which included a survey, in-depth interviews, and focus group discussions (FGDs). The survey was conducted with 593 fathers who have at least one child of age 6-23 months. Four FGDs were conducted with fathers, and 21 in-depth interviews were conducted with fathers, mothers, and community health workers. The survey data were entered into Epi data software version 1.4.4.0 and statistical analysis was performed using SPSS software version 20. Bivariate and multivariate logistic regression analyses were performed and statistical significance was considered at *p* < 0.05. All interviews and FGDs were transcribed, coded, categorized, and analyzed using open code software version 4.0.2.

**Results:**

Of the total sample of 593, 50.9% of the fathers in the study were involved in their children’s complementary feeding practices. Fathers with better household income (AOR = 1.56; 95% CI: 1.09, 2.22) and good perception of child complementary feeding practice (AOR = 1.79; 95% CI: 1.28, 2.52) were more likely to be involved in their children’s complementary feeding practice. The majority of the fathers had better knowledge about the recommended complementary feeding practices.

**Conclusions:**

Income-generating activities and behavioral change communication for fathers should be encouraged to improve their involvement in child feeding. Community-based nutrition programs should also give due attention to increasing the involvement of fathers.

## Background

Infant and young child feeding (IYCF) is a critical practice for child health and survival [1]. IYCF practices directly affect the nutritional status of infants and young children and, ultimately, impact child survival [2]. As part of IYCF practice indicators, timely introduction of complementary foods, frequency of feeding, and quality of diet make up appropriate complementary feeding practices. Infants should receive complementary foods from 6 months of age onwards [3]. Parents, particularly mothers, are primarily involved in IYCF practice [4, 5].

Parents are the primary agents to enable children to grow into healthy, responsible, and mature adults. As a parent, the contributions of fathers to their children’s development, health, and well-being are important [6]. Studies in Canada and Texas, USA showed that the role of fathers in IYCF practices was crucial in shaping children’s nutritional status along with mothers’ feeding practices taken into account [7, 8].

A mother’s decision to breastfeed and the duration of breastfeeding depends on different factors; among them is the support of the father or male partner [9]. Without supporting fathers to play a positive role in child feeding, interventions designed to promote child feeding are less likely to be effective [10]. Fathers have an important role in promoting their child’s social and emotional development and might also be a useful strategy in promoting fathers’ involvement [11]. Different studies reported that fathers’ involvement in child feeding is affected by the level of socioeconomic status, family structure, cultural conditions, religion, fathers’ knowledge and practice of child feeding, and maternal occupation [12–14].

The IYCF practice has been the emphasis of nutritional policies in many developing countries to address child malnutrition and improve the growth, health, and development of children [2]. Despite the role of fathers in IYCF practice, their involvement in child complementary feeding has rarely been studied and literature gives more attention to the role of mothers. Therefore, this study aimed to assess the level of fathers’ involvement in complementary feeding of children and to identify factors associated with it in the study area.

## Methods

### Study design and setting

A community-based cross-sectional survey, in-depth interviews and FGDs were conducted from February to March 2019 in Damot Woyde district, Southern Ethiopia. The district is located 313 km south of the Ethiopian capital, Addis Ababa through Butajira Halaba road. In 2019, the Ethiopian Central Statistical Agency (CSA) projected the total population of the district to be 113,823; of which 50,896 were children below two years of age.

### Population and sampling

For the survey, the following assumptions were used to calculate the sample size of 634; the expected prevalence of 50% for the involvement of fathers in complementary feeding practice, 95% confidence level, 5% margin of error, design effect of 1.5, and 10% non-response rate. A total of 593 fathers completed the questionnaire in the survey, making the response rate 93.5%. Fathers who had at least one child in the age group of 6–23 months were the source population. A systematic random sampling method was applied to select fathers from selected kebeles (smallest administrative units). Fathers paired with their children were included in the study. First, 634 households were selected systematically from 6 kebeles that were randomly selected out of 25 kebeles. The total sample was allocated to these 6 kebeles proportional to population size. From each household, one father was recruited to complete the survey questionnaire. In households where there was more than one eligible father lottery method was applied to select one father.

In-depth interviews and FGDs were conducted to complement the survey data. For the in-depth interviews 5 fathers, 10 mothers, and 6 community health workers were recruited conveniently by the first author from the kebeles where the survey was conducted. The number of participants in the in-depth interviews was decided based on point of saturation where there was no new information emerging from the last interviews. Similarly, 4 FGDs were conducted with fathers and mothers that were recruited conveniently by the community health workers from the community. There were a total of 61 participants in the in-depth interviews and FGDs.

### Data collection

A structured questionnaire composed of socio-demographic characteristics and questions that measure the father’s knowledge, attitude, and involvement in complementary feeding practice was prepared in English and translated into the local language. Six Bachelor of Science degree holder research assistants (RAs) who speak the local language and had previous data collection experience administered the questionnaire. The RAs were also provided with additional training for two days on the different modules of the questionnaire, participants’ selection, and ethics. The questionnaire was pretested before conducting the main survey with a sample of 32 fathers in a community with similar characteristics to the actual study population but not part of the main study.

The first author, fluent in the local language, conducted all in-depth interviews using a semi-structured interview guide. Focus group discussions were conducted with fathers and mothers, and 3 RAs facilitated the FGDs. The RAs were gender-matched with the FGD participants. Both in-depth interviews and FGDs were recorded by the digital audio recorder and on average the interview took 45 min whereas the FGDs took 60 min.

### Measurements

Fathers were asked six questions to measure their involvement in complementary feeding practice. The questions had five Likert scale responses; always, often, sometimes, rarely, and never. A single variable was established by creating a total score from the six questions. Those who scored above the mean in the total score were labeled as having “fair involvement”, whereas those who scored the mean or below in the total score were labeled as having “Poor involvement”.

Similarly, to assess fathers’ knowledge about complementary feeding, additional six Likert scale questions with five Likert scale responses were administered. Fathers with a score above the mean were labeled as having “better knowledge”, whereas those with a score of the mean or below the mean were categorized as having “poor knowledge”.

Fathers’ perception towards involvement in child complementary feeding was measured using thirteen Likert scale questions. Fathers with a score above the mean were labeled as having “better perception”, whereas those with a score of the mean or below the mean were categorized as having “poor perception”.

### Data analyses

The survey data were entered into Epi data software version 1.4.4.0 and exported to SPSS version 20 for analysis. Descriptive statistics were conducted for all variables. Normality was checked for quantitative continuous variables. We utilized binary logistic regression analysis since the outcome variable had binary outcomes. Exposure variables with *p*-value < 0.25 during bivariate analysis were considered for multivariate analysis. Multivariate logistic regression analysis was done to control for potential confounders and identify predictors of the outcome variable. Audio-recorded files of the FGDs and in-depth interviews were transcribed verbatim, translated into English, and coded. The codes were generated into common themes and sub-themes. Open code software was used to assist data organization and analysis.

## Results

### Socio-demographic characteristics

Out of 593 fathers who completed the survey, 276 (46.5%) were in age between 35–44 years and the mean age of the study participants was 36.1(SD = 2.2). Most (576, 97.1%) of the fathers were married and 421 (71.0%) were protestant Christian religion followers. The majority (563, 94.9%) of the fathers were from Wolaita ethnic group and 177 (29.8%) attended at least primary education. More than half (347, 58.5%) of the fathers were farmers (Table 1).

**Table 1:** Socio-demographic characteristics of study participants in Damot Woyde district in South Ethiopia, February 2019

	**Variables(*n* = 593)**	**Frequency**	**Percent**
**Age**	≤ 30	146	24.6
	31–44	324	54.6
	≥ 45	123	20.7
**Marital status**	Currently Married	576	97.1
	Currently Unmarried^a^	17	2.9
**Religion**	Protestant	421	71.0
	Orthodox	129	21.8
	Others^b^	43	7.2
**Ethnicity**	Wolaita	563	94.9
	Others^c^	30	5.1
**Educational status**	No formal education	287	48.4
	Primary education (1–8)	177	29.8
	Secondary and above	129	21.8
**Occupation**	Farmer	347	58.5
	Daily laborer	67	11.3
	Merchant	112	18.9
	Employed^d^	67	11.3
**Average monthly income**	< 500	321	54.1
	≥ 500	272	45.9

^a^Separated, Divorced, Widowed ^b^Muslim, Catholic ^c^Kembata, Hadiya, Amhara

^d^Non-governmental organization employee, Government organization employee

### Fathers’ knowledge about complementary feeding practice and attitude toward their involvement

More than half (357, 60.2%) of the fathers had better knowledge about child complementary feeding practice and 278(46.9%) had a better perception of fathers’ involvement in child complementary feeding (Table 2).

**Table 2:** Knowledge of fathers about complementary feeding and perception about fathers’ involvement in complementary feeding in Damot Woyde district in South Ethiopia, February 2019

Variables(*n* = 593)	Frequency	Percent
**Knowledge of complementary feeding**		
Better	357	60.2
Poor	236	39.8
**Perception about fathers’ involvement in complementary feeding practice**		
Better	278	46.9
Poor	315	53.1

Almost all study participants of the in-depth interview and FGDs reported that they know what complementary feeding is and when to start it. Most mothers and fathers believe that complementary food should be initiated at the age of 6 months.

A 35-year-old father said; “…*at the age of six months children should start complementary food.”* A 22-year-old mother also reported this and she said; “…w*e start complementary food at the age of 6 months…”.*

### The proportion of fathers’ involvement in child complementary feeding and its predictors

In this study, the proportion of fathers’ involvement in child complementary feeding practice was 50.9%. Fathers with a better household income had a 56% higher chance of being involved in their child’s complementary feeding practice (AOR = 1.56; 95% CI: 1.09, 2.22). Fathers with better perception towards fathers’ involvement in child complementary feeding were 79% more likely to involve in the practice compared to their counterparts (AOR = 1.79; 95% CI: 1.28, 2.52) (Table 3).

**Table 3:** Bivariate and multivariable regression analysis on fathers’ involvement in child complementary feeding in Damot Woyde district in South Ethiopia, February 2019

Variables (*n* = 432)	Fathers’ involvement		COR (95% CI)	AOR (95% CI)
	**Poor involvement**	**Better involvement**		
**Religion**				
Protestant	209(49.6%)	212(50.4%)	1	1
Orthodox	55(42.6%)	74(57.4%)	1.33(.89, 1.97)	1.27 (.84, 1.91)
Others	27 (62.8%)	16(37.2%)	.58(.31, 1.12)	.58 (.29, 1.15)
**Ethnicity**				
Wolaita	272(48.3%)	291(51.7%)	1	1
Others	19(63.3%)	11(36.7%)	.54(.25, 1.16)	.52(.23, 1.16)
**Education**				
No formal education	132(46.0%)	155(54.0%)	1	1
Primary (1–8)	94(53.1%)	83(46.9%)	.75(.52, 1.09)	.74(.50, 1.10)
Secondary and above	65(50.4%)	64(49.6%)	.84(.55, 1.27)	.65 (.42, 1.03)
**Income**				
< 500	169(52.9%)	152(47.4%)	1	1
≥ 500	122(44.9%)	150(55.1%)	1.37(.99, 1.89)	1.56(1.09, 2.22)**
**Fathers’ knowledge of complementary feeding**				
Poor knowledge	108(45.8%)	128(54.2%)	1	1
Better knowledge	183(51.3%)	174(48.7%)	0.80(0.58, 1.11)	.75(.53, 1.05)
**Fathers’ perception of the involvement**				
Poor perception	175(55.6%)	140(44.4%)	1	1
Better perception	116(41.7%)	162(58.3%)	1.74(1.26,2.42)	1.79(1.28, 2.52)**

^**^Statistically significant at *p* < 0.05

Most of the FGD and in-depth interview participants reported that most fathers do not take part in child feeding. The community has defined the role of fathers and mothers in child feeding and most fathers believe that involving in child feeding is not their role.

A 37-year-old male FGD participant reported:

“*What we are practicing now is what we have learned from our parents. Fathers in the past had no trend of involving in child feeding. …except for providing financially, fathers are not involved in food preparation and child feeding. This is considered as the responsibility of mothers.*”

A community health worker also reported that fathers rarely involve in child feeding.

“*…we don’t see fathers being involved in child feeding. Since we conduct a house-to-house visit in the community, we observe that fathers’ involvement in child feeding is so poor.*”

On the other hand, few fathers involve in child feeding practice. It was reported that there is a change in the attitude of fathers and they started to involve in child feeding though their contribution is far less than the mothers.

A 29-year-old mother reported:

“*Even though they are few in number, there are fathers who involve in child feeding. …fathers started to involve in child feeding though mother’s contribution is great when compared with that of fathers’.*”

## Discussion

In this study, the proportion of fathers’ involvement in child complementary feeding was found to be 50.9%. It was found that household income and fathers’ perception of their involvement were the factors associated with fathers’ involvement in child complementary feeding. It was also found that the majority of the fathers have good knowledge about the recommended complementary feeding practices.

The proportion of fathers’ who involve in complementary feeding of children was found to be 50.9%. This finding is comparable with a similar study conducted in Australia where 42% of the fathers were involved in organizing meals, 60% involve in deciding what kinds of foods their children should eat, and 50% were involved in how much food their child should be offered [6]. A study conducted in Ghana reported the overall level of fathers’ involvement in childcare and feeding to be 63.5% [15]. Another study conducted among first-time fathers reported that 73% of fathers were involved in IYCF practice [16]. This difference might be attributed to the fact that all of the study participants were first-time fathers in the above-mentioned study. The study was also conducted in an urban area, unlike the current study where both urban and rural residents were included. The sample size utilized was also small and the study participants were selected purposively unlike the report of the current study.

The involvement of fathers in child complementary feeding was 56% higher for those fathers with a household income of ≥ 500 ETB compared to their counterparts. Fathers who can provide economically are more likely to engage with and nurture their children [17].

In this study, fathers with a good perception of fathers’ involvement in child complementary feeding had a higher chance of involving in child feeding. This is in agreement with a study conducted in Madagascar [18]. Fathers with a better perception may view their roles more than being a breadwinner and involved in their children’s issues and participating in household and child-centered tasks [19]. Their perception might have come intentionally while they were looking for ways they could help their children to grow healthier.

The majority of fathers have good knowledge about the recommended complementary feeding practices. This finding is in agreement with another study conducted in Rwanda where men have a good degree of basic knowledge about key nutrition messages [20]. This might be because the health extension program works within the community to deliver the promotion of appropriate child-feeding practices and other health-related messages. It was found in this study that it is difficult for fathers to involve fully in child care because they stay most of the time outside the home for income-generation activities.

In this study, it was reported that about half of the fathers’ who participated in the study did not involve in complementary feeding of their children. The traditional gender roles, in which the mother is seen as primarily responsible for child care, might have limited fathers’ involvement in child feeding. This is supported by another study conducted in Malawi and Madagascar where fathers provided financial and material support and verbal encouragement to their spouses [18, 21]. This traditional definition might have influenced their involvement level in child complementary feeding practices.

## Conclusion and recommendations

The study showed that only one in two fathers was involved in their child’s complementary feeding. Fathers’ involvement in child complementary feeding practice was found to be affected by household income level and perception about fathers’ involvement in child complementary feeding. Therefore, community development programs and income-generating activities should be promoted. Community health workers should work to improve the perception of fathers on their involvement in child feeding. Policymakers and health planners should also optimize programs of community-based nutritional promotion programs by giving due attention to fathers.

### Limitations of the study

We did not include children’s age and gender to see whether there is an association or not with the involvement of fathers. We have no data on child complementary feeding practices and wealth index.

## Data Availability

The data set for this study is available with the corresponding author which can be accessed on reasonable request by writing to tasamsona@gmail.com.

## Abbreviations

AOR: Adjusted Odds Ratio
CF: Complementary Feeding
CI: Confidence Interval
COR: Crude Odds Ratio
DE: Design Effect
EDHS: Ethiopian Demographic and Health Survey
EFY: Ethiopian Fiscal Year
ETB: Ethiopian Birr
FGD: Focus Group Discussion
IYCF: Infant and Young Child Feeding
SPSS: Statistical Package for Social Science
UNICEF: United Nations Children’s Fund
WHO: World Health Organization
